# A Vision Transformer-Based Deep Learning Framework for Patient-Level Classification of Acute Pancreatitis and Normal Pancreas Using Computed Tomography

**DOI:** 10.3390/diagnostics16081152

**Published:** 2026-04-13

**Authors:** Gürkan Güneri, Elif Kır Yazar, Mesut Furkan Yazar, Kadir Çorbacı, Mehmet Süleyman Yıldırım, Emre Dandıl

**Affiliations:** 1 Department of General Surgery, Faculty of Medicine, Bilecik Şeyh Edebali University, Bilecik 11100, Türkiye; kadir.corbaci@bilecik.edu.tr; 2 Department of Radiology, Bilecik Training and Research Hospital, Bilecik 11100, Türkiye; 3 Department of Computer Technology, Söğüt Vocational School, Bilecik Şeyh Edebali University, Söğüt, Bilecik 11600, Türkiye; 4 Department of Computer Engineering, Faculty of Engineering, Bilecik Şeyh Edebali University, Bilecik 11100, Türkiye; emre.dandil@bilecik.edu.tr

**Keywords:** acute pancreatitis, computed tomography (CT), deep learning, vision transformer (ViT), classification, patient-level diagnosis

## Abstract

**Background/Objectives**: Acute pancreatitis (AP) is a significant inflammatory pancreatic disease with high morbidity and mortality rates that requires early and accurate diagnosis. In this study, a deep learning-based classification system is developed and evaluated for the automatic classification of AP and normal pancreas from contrast-enhanced CT images, with a focus on patient-level assessment to enhance clinical applicability. **Methods**: A study-specific dataset is created for the study in CT images from 183 patients (103 normal and 80 with AP). To prevent data leakage and objectively evaluate model performance, the dataset is divided into training and test sets based on patient-level data. Convolutional neural network (CNN)-based architectures, such as ResNet50, EfficientNet, and ConvNeXtV2, are compared with Transformer-based architectures, such as Swin Transformer (Swin) and Vision Transformer (ViT). **Results**: In slice-level analysis, all models achieve high performance. Swin shows the highest accuracy (84.06%), and ViT revealed the most balanced performance with an F1-score of 82.90%. In the more clinically significant patient-level evaluation, the ViT model outperforms all others with an accuracy of 89.19%, an F1-score of 86.67%, and an area under the curve (AUC) of 0.946. The ViT model’s high AUC and recall values demonstrate its ability to reliably distinguish between AP and normal pancreas classes, even under different threshold values. **Conclusions**: These results suggest that transformer-based architectures can extract stronger and more reliable feature representations from pancreatic CT images due to their capacity to model global contextual features. Furthermore, the patient-level evaluation approach enables the model to generate results that are more compatible with clinical decision-making processes, thereby enhancing its usability in real clinical settings. In conclusion, the proposed ViT-based approach is a promising method for diagnosing acute pancreatitis.

## 1. Introduction

The pancreas is a retroperitoneal organ with a complex anatomical structure extending transversely from the duodenal curve to the splenic hilum. This structure is anatomically divided into four sections: the head, the uncinate process, the body, and the tail [1]. Contrast-enhanced computed tomography (CT) is the primary imaging method for the pancreas. It enables a detailed evaluation of the pancreatic parenchyma and clearly visualizes the surrounding anatomical relationships thanks to its high resolution [2,3]. A normal (healthy) pancreas is typically seen on CT as a structure with homogeneous soft tissue density, smooth contours, and relatively heterogeneous contrast enhancement [4]. However, its morphology and density characteristics may vary depending on patient-related factors and the imaging phase. Pathological processes affecting the pancreas often cause changes in its CT appearance, such as focal or diffuse enlargement, irregular contours, heterogeneous density, and involvement of adjacent tissues [5]. In particular, inflammatory conditions may be accompanied by increased density and heterogeneity of peripancreatic adipose tissue, fluid collections, vascular complications, and parenchymal heterogeneity. These findings are the basis for radiological evaluations in clinical practice [6].

Acute pancreatitis (AP) is defined as inflammation of the pancreatic parenchyma [7]. This inflammation can be caused by several factors, including chronic alcoholism, gallstones, hypertriglyceridemia syndromes, drugs, and chemical agents. AP is one of the most common gastrointestinal causes of hospitalization worldwide. It presents with a wide range of clinical courses, from mild, self-limiting inflammation to severe disease associated with permanent organ failure and high mortality rates [8,9]. Mortality rates of 30–40% have been reported, particularly among patients who develop organ failure [10]. The diagnosis of AP is based on abdominal pain, predominantly in the epigastric region, amylase and lipase levels more than three times higher than normal, and imaging findings (peripancreatic fluid, pancreatic inflammation), with at least two of these criteria being positive [8,11].

The deep location of the pancreas, its proximity to other organs, and the inflammatory changes in the pancreas and surrounding tissues following AP led to blurring of the pancreatic boundaries. This makes it difficult to assess necrosis and peripancreatic involvement rates, resulting in interobserver variability in radiological severity classification [12,13]. Therefore, difficulties may arise in reporting CT scans and in determining the severity of the disease. An early and accurate diagnosis is critical for determining appropriate treatment strategies, preventing complications, and reducing mortality rates. Reliably differentiating AP from normal pancreatic tissue plays a significant role in clinical decision-making. Accurate and early classification of AP relative to normal pancreas tissue is crucial for predicting the disease’s clinical course and for guiding clinical decisions, such as admission to the intensive care unit, monitoring strategy determination, and intervention timing.

In AP, the revised Atlanta classification is used clinically, while the Balthazar classification and the CT severity index are used radiologically [8,14]. However, both types of scoring rely heavily on subjective evaluation, which can lead to discrepancies in assessment among clinicians and radiologists, particularly in the early stages of the disease or with borderline patients. Furthermore, manually evaluating CT images depends on the specialist’s experience and can present diagnostic difficulties, particularly in cases of early-stage acute pancreatitis or cases with subtle morphological changes. Additionally, manually analyzing high volumes of image data is time-consuming and increases the clinical workload.

Previous studies on the analysis of pancreatic images mostly focus on pancreatic segmentation or pancreatic cancer detection [15,16,17,18,19]. Recently, artificial intelligence-based approaches have been used to improve the standardization of classification in AP [20,21]. In addition, applications based on deep learning technologies have significantly advanced the analysis of medical images in recent years [22,23,24]. Moreover, deep learning models applied to CT images have shown promising results in differentiating between mild and severe disease, either alone or in combination with clinical and laboratory parameters from radiological images [25,26]. However, studies on the automatic detection of AP and the classification of AP and normal pancreas are limited. Many current studies focus on CNN-based deep learning methods, but the effectiveness of ViT-based architectures for this purpose has not been sufficiently addressed. In addition, while many studies evaluate on a slice-by-slice basis, only a few studies address the more clinically meaningful patient-level classification approach. Patient-level classification provides results that are more consistent with the clinical diagnostic process because it enables a holistic evaluation of the information in the entire CT scan [27]. This study proposes a transformer-based deep learning model for the automatic classification of AP and normal pancreas from CT images. The model effectively learns local and global features from CT images for patient-level classification. The main contributions of this study are summarized below:

i. Automatic classification of AP and normal pancreas from CT images using a ViT-based deep learning model.
ii. A patient-level classification approach that is more meaningful in terms of clinical applications.
iii. A comprehensive comparison of the performance of the transformer-based model with that of other state-of-the-art deep learning architectures.
iv. Improving classification performance by effectively learning local and global features from CT images.
v. Demonstration of the usability of artificial intelligence–based decision support systems in diagnosing AP and contributing to clinical applications.

## 2. Materials and Methods

**Figure 1:**
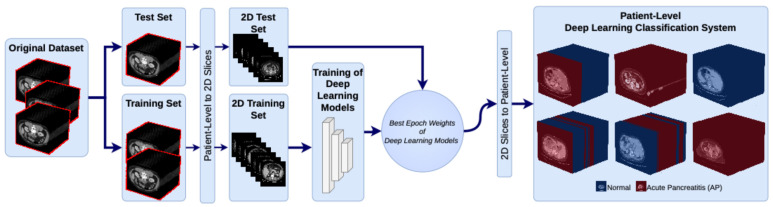
Overall workflow of the proposed deep learning framework for patient-level classification of acute pancreatitis and normal pancreas from CT scans.

Figure 1 shows the general workflow of the proposed deep learning-based system for the classification of AP and normal pancreas from CT images. The system consists of four stages: data preparation, model training, model selection, and patient-level classification. First, the original CT dataset is split into training and test sets on a patient-by-patient basis. In order to avoid data leakage and ensure a reliable evaluation of model generalizability, patient-level data splitting was performed, such that all CT slices belonging to a given patient were assigned exclusively to either the training or the test set. Data leakage occurs when information from the test set is unintentionally used during training, resulting in overly optimistic and biased performance estimates. In medical imaging studies, this commonly occurs when images or slices from the same patient are included in both the training and test sets. This approach provides a more reliable performance evaluation for clinical practice. Next, patient-level CT volumes are converted into two-dimensional (2D) image slices. This process results in separate 2D image datasets for the training and test sets. This step enables deep learning models to extract image-based features more effectively. In the third stage, different deep learning models are trained using the created 2D training set. In this study, CT slices from the same patient were strictly assigned to either the training set or the test set. This patient-level separation ensures that the model is evaluated on completely unseen patients, thereby preventing data leakage and providing a realistic assessment of generalization performance. Model performance is evaluated in this stage using various state-of-the-art deep learning architectures, primarily the transformer-based model. After training, the models that yielded the best results according to performance metrics are selected for use in the classification system. In the final stage, the best-trained model is used to classify 2D CT slices from the test set. The classification results obtained for each slice are then combined on a patient-by-patient basis to determine the final classification result for each patient. This approach increases diagnostic accuracy by enabling a holistic evaluation of information obtained from individual image slices. Consequently, the proposed deep learning-based classification system automatically categorizes patients as having AP or a normal pancreas.

### 2.1. Dataset

The pancreatic CT image dataset used in this study was obtained retrospectively from Bilecik Training and Research Hospital. It was created from images of patients who came to the hospital and had abdominal CT scans between 1 December 2020 and 1 December 2025. CT images of patients diagnosed with AP and treated as inpatients or outpatients were included in the AP category. The normal group consisted of images of individuals without an AP diagnosis who underwent abdominal CT imaging for various clinical reasons. All images were evaluated on a patient-by-patient basis, and clinical diagnoses were used to create the dataset. Ethical approval for this study was obtained from the Non-Interventional Clinical Research Ethics Committee of Bilecik Seyh Edebali University (with meeting number 9, decision number 12, 30 October 2024). The study was conducted in accordance with the principles of the Helsinki Declaration, and all data were anonymized to protect patient confidentiality. In this study, CT examinations for the dataset were performed using a 64-detector multi-slice CT system (Revolution EVO) manufactured by GE Healthcare. The device was manufactured by GE Hangwei Medical Systems Co., Ltd., Beijing, China, for GE Healthcare. Intravenous contrast-enhanced abdominal scans were routinely acquired at a slice thickness of 1.25 mm to enable thin-slice reconstructions. In addition, the CT scans acquired during the portal venous phase, 60–70 s following contrast injection. This phase was selected as the standard protocol because it provides optimal contrast enhancement for evaluating pancreatic and intestinal pathologies. The system operates with a Performix 40 Plus X-ray tube (GE Healthcare manufactured by GE Hangwei Medical Systems Co., Ltd., Beijing, China) and the manufacturer’s standard imaging software. All examinations were performed in accordance with the manufacturer’s protocols and international radiation safety standards. The original CT images have a resolution of 512 × 512 pixels. These parameters were used to ensure optimal visualization of the pancreatic tissue and surrounding anatomical structures.

As shown in Table 1, the dataset consists of CT images from a total of 183 patients. Of these, 80 are in the AP group and 103 are in the normal group. In terms of gender distribution, 45 (56.3%) of the patients in the AP group are women and 35 (43.8%) are men, while 35 (34.0%) of the patients in the normal group are women and 68 (66.0%) are men. Overall, the dataset includes 80 women (43.7%) and 103 men (56.3%). Regarding age distribution, the average age of female patients in the AP group was 59.8 ± 17.0 years, while the average age of male patients was 56.0 ± 13.5 years. In the normal group, the average age of female patients was 44.4 ± 12.1 years and the average age of male patients was 39.9 ± 13.8 years. The average age across the entire dataset was 53.1 ± 16.8 years for women and 45.4 ± 15.7 years for men. The average age of patients in the AP group was 57.9 years, while the average age of patients in the normal group was 42.16 years. Overall, the mean age of patients in the AP group was higher than that of the normal group. This finding is consistent with clinical observations that support the idea that AP is more prevalent in older age groups. Due to its inclusion of patient groups with different age and gender distributions, this dataset provides a suitable basis for evaluating the generalizability of the proposed deep learning model. Furthermore, detailed pancreatic tissue analysis was possible thanks to high-resolution CT images and thin slice thickness. This enabled deep learning models to effectively learn both local and global image features.

The biochemical and hematological parameters of patients in the AP group, based on routine laboratory test results collected at hospital admission, are presented in Table 2. These parameters include clinically important biomarkers for the diagnosis of AP, assessment of severity, and determination of systemic inflammatory response. The analyzed parameters included pancreatic enzymes (amylase and lipase), liver function markers (GGT), kidney function markers (urea and creatinine), hematological parameters (white blood cell count, neutrophil count, platelet count, hemoglobin, and hematocrit), and C-reactive protein (CRP), an inflammation marker. In female patients in the AP group, the mean serum amylase level was measured as 1578.05 ± 958.65 U/L, and the mean lipase level was 3650.51 ± 2523.62 U/L. In male patients, the mean amylase level was determined as 1338.56 ± 1182.88 U/L and the lipase level as 2871.63 ± 2829.78 U/L. Both amylase and lipase levels were significantly higher than the normal reference ranges in both genders, which supports biochemical confirmation of AP. Liver function markers revealed that gamma-glutamyl transferase (GGT) levels were, on average, 280.43 ± 414.39 U/L in female patients and 258.21 ± 220.12 U/L in male patients. Regarding renal function, urea levels averaged 32.09 ± 10.37 mg/dL and 30.74 ± 10.19 mg/dL in female and male patients, respectively. Creatinine levels averaged 0.77 ± 0.18 mg/dL and 0.96 ± 0.16 mg/dL in female and male patients, respectively. Hematological parameters revealed that white blood cell (WBC) counts averaged 12.43 ± 4.85 ×10^3^/µL and 12.91 ± 4.80 ×10^3^/µL in female and male patients, respectively. The average neutrophil count was 10.26 ± 4.91 ×10^3^/µL in female patients and 10.35 ± 4.77 ×10^3^/µL in male patients. These values indicate the presence of an inflammatory response associated with AP. The platelet counts averaged 277.95 ± 68.98 ×10^3^/µL in female patients and 245.62 ± 75.43 ×10^3^/µL in male patients. The hemoglobin level averaged 13.00 ± 1.89 g/dL in female patients and 14.43 ± 2.04 g/dL in male patients. Hematocrit values were found to be 38.70 ± 5.01% in female patients and 42.32 ± 5.18% in male patients, on average. CRP, a key biomarker of inflammation, was measured at an average of 9.25 ± 12.88 mg/L in females and 32.51 ± 50.79 mg/L in males, supporting the presence of an inflammatory response in the AP group. These laboratory findings demonstrate that the AP group in the dataset consists of clinically validated and biochemically supported cases. In particular, the high levels of pancreatic enzymes and increased inflammatory markers reveal that the dataset reliably represents the diagnosis of AP. This enables the proposed deep learning model to be trained on reliable, clinically validated data, thereby increasing the model’s clinical applicability.

**Figure 2:**
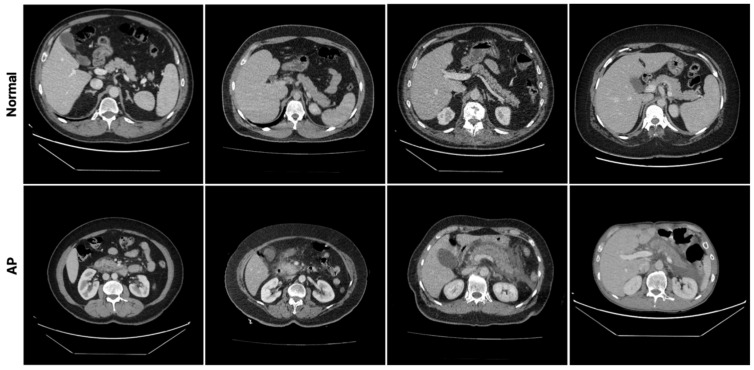
Representative CT images showing examples from AP and normal pancreas groups included in the dataset.

The CT scans included in the study were reviewed independently by two radiology specialists who were different from the reporting radiologist. This review was performed in a blinded manner to confirm the diagnoses. The evaluation was performed based on the full CT volume, taking into account all slices. To illustrate the visual characteristics of the dataset and the morphological differences between AP and normal pancreatic tissue, representative sample CT slices from both groups are presented in Figure 2. The figure presents axial CT slices from four patients in the AP group and four patients in the normal pancreas group. These images represent the anatomical variations, tissue density differences, and inflammatory changes present in the dataset. Examining the images from the AP group reveals morphological findings characteristic of AP, including heterogeneous density distribution in pancreatic tissue, inflammation around the pancreas, irregular tissue boundaries, and increased density in surrounding soft tissues. In contrast, images from the normal pancreas group show more homogeneous pancreatic tissue density, regular tissue boundaries, and an absence of inflammatory findings in the surrounding tissues. The sample images demonstrate that the dataset encompasses diverse visual characteristics of both pathological and normal anatomical structures. This diversity significantly improves the ability of the proposed deep learning model to distinguish between normal and pathological pancreatic tissues. Furthermore, including different anatomical and pathological variations in the dataset that represent real clinical scenarios enhances the model’s generalizability in clinical settings.

### 2.2. Proposed Methodology

This study compares the classification performance of different deep learning architectures in the automatic classification of AP and normal pancreas from CT images using both CNN-based and transformer-based deep learning models. Specifically, the CNN-based models ResNet50, EfficientNet, and ConvNeXtV2 architectures were used, while the transformer-based models Vision Transformer (ViT) and Swin Transformer (Swin) architectures were employed. The significance of the proposed methodology involves in demonstrating the effectiveness of the ViT architecture in evaluating the automatic classification performance of AP and normal pancreas.

The ResNet50 CNN architecture mitigates the gradient loss issue that arises when training deep networks, thanks to its residual connection mechanism [28]. The model’s skip connections allow the input to pass directly through certain layers to subsequent stages, enabling the network to focus solely on residual learning [29]. The ResNet50 structure, consisting of 50 trainable layers, minimizes computational cost while maximizing feature extraction capacity thanks to bottleneck blocks containing 1 × 1, 3 × 3 and again 1 × 1 convolution layers [30]. Another CNN model, EfficientNet (B0), provides high accuracy and computational efficiency by balancing the scaling of model depth, width, and resolution [31]. Built on mobile convolution blocks using depthwise separable convolutions, the EfficientNet architecture can achieve similar or more successful results with a much lower number of parameters than traditional CNNs [32].

ConvNeXtV2 is another deep learning architecture used in the study. It is inspired by modern transformer design principles and significantly improves the performance of CNN architectures [33]. By adopting the training strategies and structural preferences of Transformer structures, the ConvNeXtV2 model generates a non-local receptive field by employing large 7 × 7 convolutional cores instead of the standard 3 × 3 filters [34]. ConvNeXtV2 is distinguished by its inverted bottleneck structure and simplified normalization layers. It is a hybrid structure that combines the ease of implementation of convolutional networks with the high representational power of transformers [35].

The Swin Base Transformer (Swin) is a transformer-based architecture that enables the efficient learning of local and global features. It accomplishes this by dividing an image into hierarchical windows and using a shifted, window-based self-attention mechanism [36]. This model processes images as non-overlapping windows and shifts the positions of these windows between layers to carry out the flow of information between neighboring regions, leading to the preservation of global context.

This study focuses on the ViT architecture specifically. The ViT is a deep learning model based on the transformer architecture that divides an image into 16 × 16 patches. It processes these patches as a sequence and learns global relationships within the image using a self-attention mechanism [37]. Unlike CNN-based architectures, the ViT model learns features directly using a self-attention mechanism, bypassing convolution entirely [38]. This allows it to model relationships between distant regions of an image more effectively and learn global contextual features more efficiently. The ViT architecture consists of three main components: the patch embedding layer, transformer encoder blocks, and the classification layer [39]. In the patch embedding layer, the input image is divided into fixed-size patches, and each patch is converted into a vector representation [40]. In the ViT architecture, the process of dividing CT slices into parts suitable for the transformer encoder is performed in the linear projection of flattened patches block [41]. During training, the classification head and the divided parts are sent to the transformer encoder. Classification and location information are added to the patches using a single linear layer in the multi-layer perceptron (MLP). In this process, the prepared vector sequence serves as the input for the transformer encoder [37]. Then, the transformer encoder processes these patch vectors using transformer encoder blocks, which consist of multi-head self-attention and MLP layers [42]. The self-attention mechanism enables the model to learn relationships between different regions of the image, creating stronger representation vectors for classification [43]. Finally, the obtained feature is transmitted to the classification layer to determine the image’s class.

**Figure 3:**
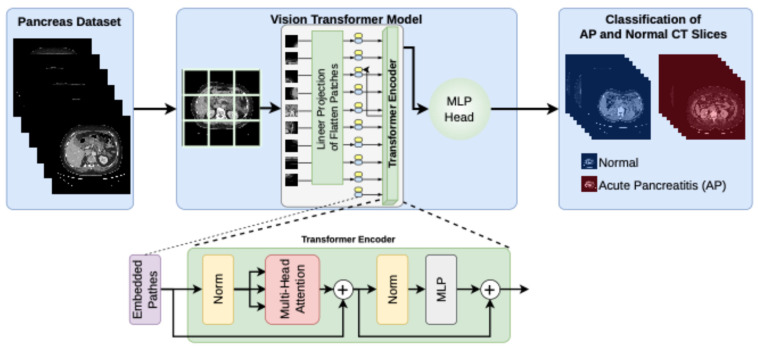
The ViT architecture proposed in this study for classification of AP and normal pancreas from pancreatic CT scans.

In this study, the ViT architecture proposed for the classification of AP and normal pancreas from pancreatic CT images is shown in Figure 3. Because the transformer-based ViT architecture effectively learns global contextual features via the self-attention mechanism, it can more accurately detect pathological changes in pancreatic tissue.

## 3. Results

In this study, a classification system based on ViT and CNN deep learning architectures was developed for the automatic classification of AP and normal pancreas from CT images. Various experimental analyses were conducted to evaluate the system’s performance comprehensively, and different state-of-the-art deep learning architectures, such as Swin, ConvNeXtV2, EfficientNet, and ResNet50, were comparatively examined. All experiments were conducted using the Python programming language (v3.11.9). The PyTorch deep learning framework (v2.6.0) was used in the development, training, and test of the deep learning models. PyTorch is a popular choice for medical image analysis studies due to its dynamic computational graphics, GPU acceleration support, and flexible model development infrastructure. Moreover, the PyTorch image models (TIMM) library (v1.0.14) was employed to facilitate the straightforward and efficient implementation of contemporary, optimized deep learning architectures.

All experimental processes were performed on a workstation running the high-performance Linux Ubuntu operating system. During training and testing, GPU acceleration was used to reduce model training time significantly. The experimental environment on the workstation featured a Gigabyte B650M motherboard and an AMD Ryzen 5–5.17 GHz processor, equipped with a total of 64 GB of DDR4 RAM, enabling efficient processing of large-scale medical image data in memory. The graphics processing unit (GPU) was an NVIDIA GeForce RTX 4070 Ti SUPER with 16 GB RAM. Thanks to GPU acceleration, training deep learning models was sped up significantly, enabling efficient processing of high-resolution CT images. Data storage was performed using a 5 TB disk with high read-write speeds.

The pancreatic dataset, which was created specifically for this study, was generated from CT images of patients with AP and normal pancreatic tissue. The dataset contains CT images from 183 patients, 103 of whom are in the normal group and 80 of whom are in the AP group. At the beginning of the data preparation process, the patients were divided into two classes according to their clinical diagnoses: normal and AP. The appearance of pancreatic tissue can be influenced by CT acquisition protocols and contrast enhancement phases, not only due to pathology, but also due to imaging conditions. In our dataset, CT scans were acquired under routine clinical conditions. Therefore, images obtained using different contrast enhancement phases may be included in both normal and AP cases. The image windowing parameters were set to a window width (WW) of 400 and a window level (WL) of 50 to ensure optimal contrast and visibility of the pancreatic tissue. These settings allow for more distinct visualization of the pancreatic parenchyma and surrounding anatomical structures, highlighting the model’s features. To prevent slice-level from the same patient from appearing in both the training and test sets and to reliably evaluate the model’s generalization ability, the dataset was split into training and test sets on a patient-by-patient basis. To avoid analyzing anatomical regions that could affect classification performance, only slices with clearly visible pancreatic tissue were selected for the analysis.

Each patient’s CT scan consists of 200 to 450 axial slices. To train deep learning models, all CT slices were converted from the original DICOM format to PNG format. In patient-level, approximately 80% of the data was allocated to the training set, and the remaining 20% was allocated to the test set. Of the patients in the normal group, 82 were in the training set and 21 were in the test set. Of the patients in the AP group, 64 were in the training set and 16 were in the test set. This distribution provides a balanced training and testing structure for both groups. When evaluated on a slice-level basis, 4929 out of 6225 (79.18%) slices in the normal group were in the training set, and 1296 out of 6225 (20.82%) were in the test set. In the AP group, out of 5542 slices, 4309 (77.75%) were in the training set, and 1233 (22.25%) were in the test set. In addition, the model training did not include demographic data or laboratory findings.

During the training process, the deep learning models were trained using only the training set. The weighting parameters that provided the best performance for each model were then determined. Afterwards, a test dataset was used to evaluate the performance of the models with these optimal weights. Model performance was analyzed at two levels. First, we evaluated slice-level classification performance, followed by patient-level classification performance, the latter of which is more suitable for clinical applications. In this study, patient-level classification was performed using an outcome-level aggregation strategy rather than a feature-level one. Specifically, the model independently classified each CT slice, and the final patient-level decision was obtained by aggregating the predictions of all slices belonging to the same patient. The aggregation was performed using a majority voting approach, whereby the final class label was determined based on the most common prediction across all slices. This approach reduces the impact of potential misclassifications at slice level and provides a more stable, clinically meaningful prediction at patient level.

In this study, both CNN-based and transformer-based deep learning architectures were used to classify AP and normal pancreas from CT images. During the experimental evaluation process, all models were trained and tested using the same dataset and the same training and test splitting strategy. The same preprocessing steps were also used for all models. In addition, training and test sets were created using a patient-level data splitting method to prevent data leakage. To ensure a fair and objective comparison of the models, common hyperparameters were defined for all architectures and the same training configuration was applied. This allows the results to more accurately reflect the system’s real-world performance in clinical settings. First, all models were trained for 100 epochs. During training, the batch size parameter was set to 64, representing the number of image slices presented to the model simultaneously in each iteration. This value optimizes training time and ensures stable model learning. The memory capacity of the GPU and the number of parameters of the model architecture were considered when determining this parameter. The learning rate, a critical hyperparameter that determines the step size used to update network weights during training, was set to 0.0001. The image size parameter determines the resolution at which the CT images given to the model are resized. To ensure comparisons across all architectures are fair, the input image size was standardized to 224 × 224 pixels. Since this study includes two classes (AP and normal pancreas), the num_classes value was set to 2. The Adam optimization algorithm, which has an adaptive learning rate update mechanism, was used to optimize the model. The cross-entropy loss function, which is commonly used for classification problems, was used as the loss function.

**Figure 4:**
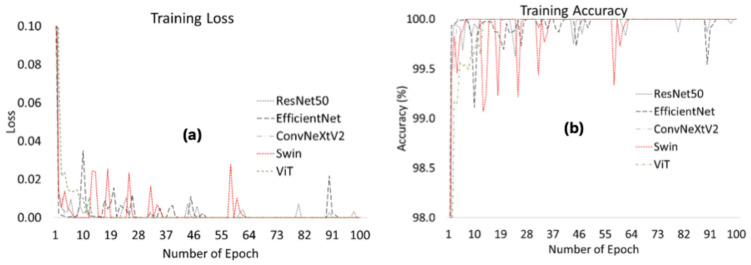
Comparison of training loss and training accuracy of deep learning models during the training process across 100 epochs. (**a**) training loss; (**b**) training accuracy.

While powerful, ViT models may struggle with overfitting and limited generalization when trained on relatively small or imbalanced medical datasets, such as pancreatic CT images. These issues are particularly relevant in medical imaging tasks, where acquiring data is difficult and class distributions may be imbalanced. In this study, MixUp was applied to dataset during the training phase. MixUp is a data augmentation technique which generates new training samples by combining pairs of input images and their corresponding labels in a linear way [44,45]. This approach enables the model to learn smoother decision boundaries and reduces the risk of memorization. For this study, MixUp was only applied during the training phase, with an alpha parameter of 0.2. The validation and test datasets remained unchanged. The aim of integrating MixUp into the training pipeline is to improve the model’s ability to capture subtle differences between AP and normal pancreatic tissue. Figure 4 shows the training loss and accuracy values obtained during the training process for the ResNet50, EfficientNet, ConvNeXtV2, Swin, and ViT models evaluated in this study. Figure 4a shows that all of the deep learning models exhibit rapid loss reduction in the early stages of training, indicating that they can quickly learn the dataset’s fundamental features. Notably, the ViT and ConvNeXtV2 models achieved lower loss values within the initial epochs of training. This demonstrates that transformer-based and modern CNN architectures can effectively learn local and global features in image data. In the later stages, the loss values of all models converge to zero and stabilize. However, some models, particularly the Swin and ResNet50 architectures, exhibited small fluctuations at certain epochs. Figure 4b shows that the training accuracy of all models quickly reaches high values. In later epochs, the models’ accuracy stabilized at high levels. Notably, the ViT and ConvNeXtV2 models converged faster in terms of training accuracy and exhibited more stable learning behavior. This indicates that these models have a high representation learning capacity. Although the EfficientNet and ResNet50 models achieved high accuracy, their convergence speeds were relatively slower compared to transformer-based models. Overall, all models successfully converged during training and effectively learned the distinctive features in the dataset.

In this study, several key metrics were used to evaluate the classification performance of deep learning models. These metrics are Accuracy, Precision, Recall, and F1-score, respectively. They are given in Equations (1)–(4). These metrics are commonly used in binary classification problems and are based on true positives (TPs), true negatives (TNs), false positives (FPs), and false negatives (FNs) in the confusion matrix. Accuracy expresses the rate at which the model correctly classifies samples and measures its overall success. Precision shows the accuracy rate of positive predictions and measures how many of the samples predicted as positive by the model are actually positive. Recall, on the other hand, shows how many of the true positive samples were correctly classified by the model. It is particularly important in cases where false negatives (FNs) are critical. The F1-score is the harmonic mean of precision and recall and is preferred in imbalanced datasets.

(1) $$Accuracy=\frac{TP+TN}{TP+TN+FP+FN}$$

(2) $$Precision=\frac{TP}{TP+FP}$$

(3) $$Recall=\frac{TP}{TP+FN}$$

(4) $$F1=2\times \frac{Precision\times Recall}{Precision+Recall}$$

**Figure 5:**
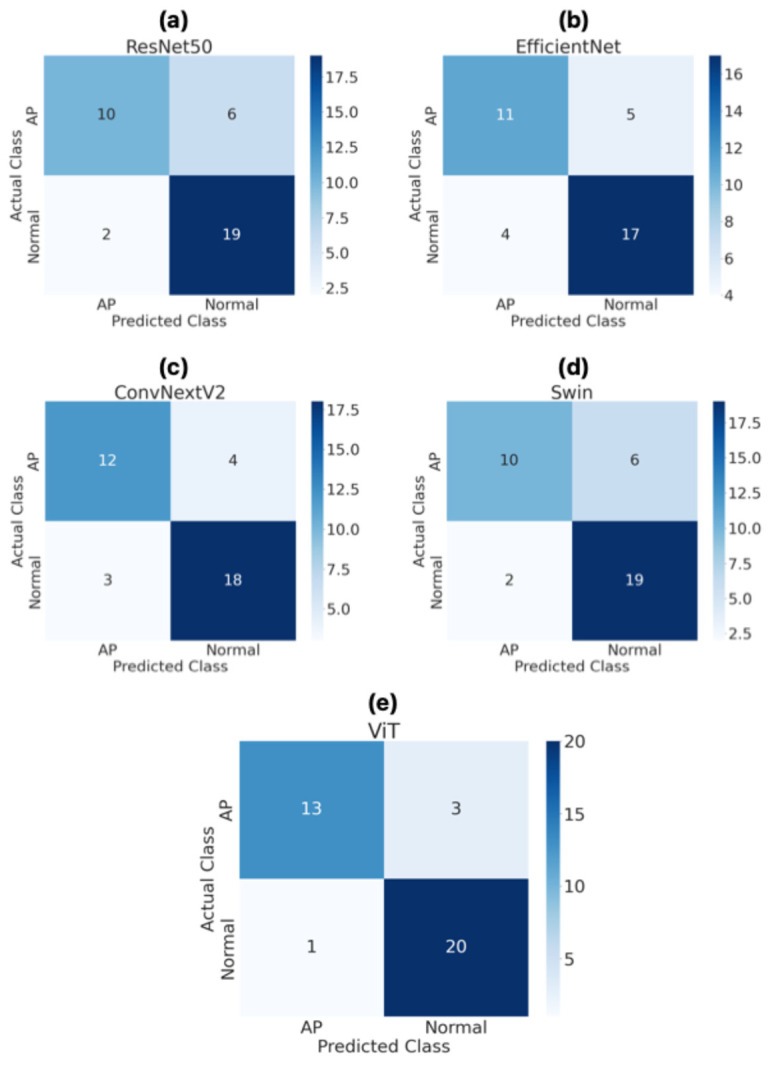
Confusion matrices for patient-level classification performance of ResNet50 (**a**), EfficientNet (**b**), ConvNeXtV2 (**c**), Swin (**d**), and ViT (**e**) models on the test dataset.

Figure 5 shows how the ResNet50, EfficientNet, ConvNeXtV2, Swin, and ViT models classify patients using confusion matrices on the test set. The analysis evaluates the models’ ability to distinguish between the AP and normal classes using clinically significant performance metrics. The ResNet50 model correctly classified 10 out of 16 patients in the AP group and incorrectly classified 6 patients as normal. It correctly classified 19 out of 21 normal patients, while incorrectly classifying 2 as AP. These results suggest that the ResNet50 model is more effective in identifying normal patients, though it has relatively lower sensitivity in detecting AP cases. The EfficientNet model correctly classified 11 patients in the AP group and incorrectly classified 5 patients. In the normal class, it achieved 17 correct classifications and 4 incorrect ones. The EfficientNet model indicated the balanced performance for both classes but limited performance in terms of overall classification accuracy. The ConvNeXtV2 model achieved 12 correct classifications and 4 incorrect classifications in the AP class and 18 correct classifications and 3 incorrect classifications in the normal class. These results demonstrate that the ConvNeXtV2 model has a more balanced accuracy rate for both the AP and normal classes. Specifically, the lower number of incorrect classifications compared to other CNN-based models indicates that the ConvNeXtV2 architecture has stronger feature representation capabilities. In addition, Swin model obtained 10 correct and 6 incorrect classifications in the AP group and 19 correct and 2 incorrect classifications in the normal group. While the Swin model performed well in identifying the normal class, it struggled to detect AP cases. This suggests that the model tends to misclassify pathological cases as normal. The ViT model, on the other hand, shown the best classification performance of all the models. It correctly classified 13 out of 16 patients in the AP class and incorrectly classified only 3 patients. In the normal class, the ViT model correctly classified 20 out of 21 patients, misclassifying only 1. These results show that the ViT model reveals the greatest sensitivity and specificity in distinguishing between the AP and normal classes.

The metric-based results presented in Table 3 reveal the performance of deep learning models evaluated on a patient-level test set in classifying AP and normal pancreas. Compared to other models, the ViT model achieved the highest results in all performance metrics, with a Precision of 92.86%, a Recall of 81.25%, an F1-score of 86.67%, and an Accuracy of 89.19%. The high Precision value indicates that the model correctly identified the vast majority of patients classified as AP. Similarly, the high Recall value shows that the ViT model is more successful than other models at identifying AP patients. The high F1-score indicates that the model has successfully achieved a balance between the Precision and Recall metrics. These results demonstrate that, thanks to its self-attention mechanism, the ViT architecture can learn pathological changes in pancreatic tissue more effectively and possesses stronger representational abilities. On the other hand, the ConvNeXtV2 model demonstrated the highest performance among the other models, achieving 81.08% Accuracy and a 77.42% F1-score. Its 75.00% Recall rate shows that it is more successful at detecting AP patients than the EfficientNet, ResNet50, and Swin models. This suggests that the ConvNeXtV2 architecture can perform more powerful feature extraction due to its modern CNN design.

**Figure 6:**
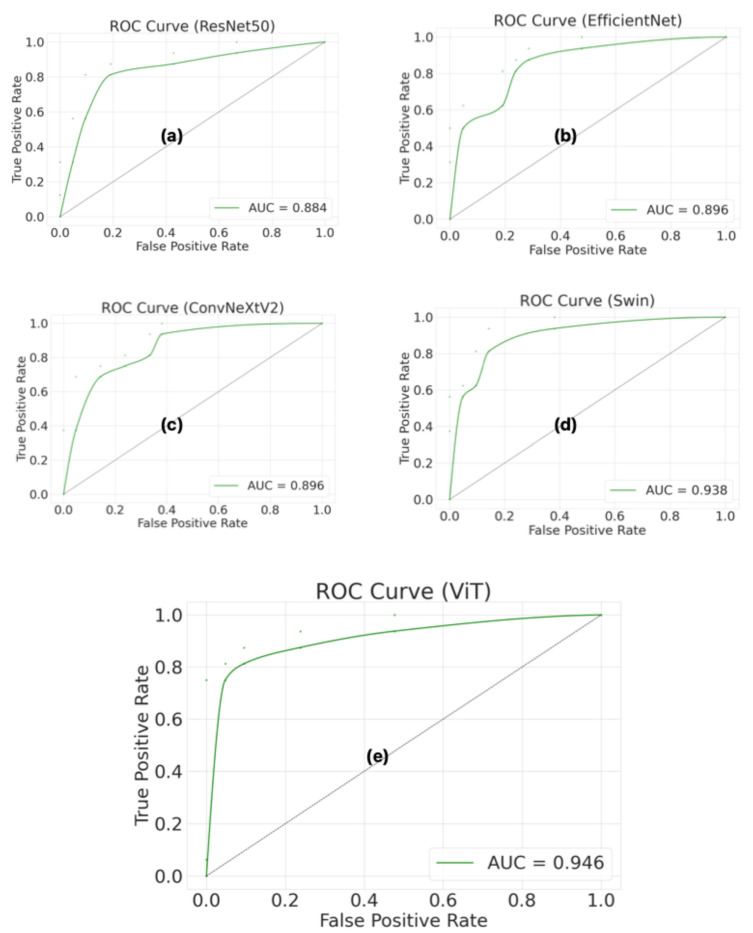
Comparison of ROC curves and AUC values of deep learning models for patient-level classification. (**a**) ROC curve for ResNet50; (**b**) ROC curve for EfficientNet; (**c**) ROC curve for ConvNeXtV2; (**d**) ROC curve for Swin; (**e**) ROC curve for ViT.

Figure 6 shows the receiver operating characteristic (ROC) curves and area under the curve (AUC) values for the ResNet50, EfficientNet, ConvNeXtV2, Swin, and ViT models, which were evaluated using a patient-level test set. The ROC curve and the AUC were used to evaluate the model’s ability to discriminate across different classification thresholds. The ViT model achieved the highest AUC value (0.946). The ViT model’s ROC curve is closest to the upper left corner, indicating that it performs better than other models in distinguishing between AP and normal pancreas classes. Furthermore, the high AUC value shows that the ViT model can provide high sensitivity and a low false positive rate at different thresholds. The Swin model indicated the closest performance to the ViT model, with an AUC of 0.938. The Swin model’s ROC curve is significantly distant from the diagonal line, indicating strong classification performance. The ConvNeXtV2 and EfficientNet models showed similar performance, with an approximately 0.896 AUC value for both. However, the ResNet50 model showed the lowest performance, with an AUC of 0.884. Additionally, the Swin model’s higher AUC value despite having the same confusion matrix results as ResNet50 indicates stronger overall classification performance. These findings confirm that the Swin model reveals more consistent and robust discrimination across all thresholds, not just at a single threshold.

In this study, a data augmentation strategy was employed to mitigate the impact of the dataset’s limited size on model performance and enhance its generalization ability. To this goal, the dataset was expanded to approximately three times its original size by applying ±20° rotation and ±20% vertical shift to the images in the training and test datasets. Examining the results presented in Table 4 reveals that transformer-based models in particular gained significantly from this process. The ViT model achieved the highest sensitivity, with a recall value of 90.90%, making it the most successful model for accurately detecting AP cases. From a clinical perspective, a high recall value is crucial for minimizing false negatives. Similarly to ViT, the Swin model stands out as another model demonstrating balanced performance, with an accuracy of 85.14% and an F1-score of 82.16%. This approach has increased the robustness of the model against different spatial variations and reduced the risk of overfitting.

A majority of deep learning studies based on pancreatic CT images focus on detecting and segmenting pancreatic tumors, as well as determining pancreatic necrosis and disease severity [46,47,48,49]. However, studies focused on classifying normal pancreases and AP are quite limited [20,50]. Of the existing studies, most use a slice-level classification approach [20]. In this study, we also evaluated the performance of deep learning models for slice-level results. According to the results presented in Table 5, the Swin model achieved the best classification performance with 93.78% of Precision, 72.10% of Recall, an F1-score of 81.52%, and 84.06% of Accuracy. In contrast, the ViT model showed balanced performance, achieving 83.75% of Accuracy and an F1-score of 82.90% in the slice-level evaluation. Among CNN-based models, ResNet50, EfficientNet, and ConvNeXtV2 showed similar performance values. However, although slice-level classification methods can provide high accuracy values from a technical standpoint, they have limitations in clinical applications. In clinical practice, diagnostic and treatment decisions are performed on a patient-by-patient basis, not on a slice-by-slice basis. Slices containing the pancreas constitute approximately 20% of abdominal CT scans [51,52]. Therefore, slice-level evaluation methods may be affected by slices other than those containing the pancreas. This may limit the direct applicability of model performance in a clinical setting. Thus, high slice-level accuracy rates do not guarantee the model’s success in the clinical diagnostic process.

## 4. Discussion

In this study, CNN and transformer-based deep learning architectures were compared for the automatic classification of AP and normal pancreas from contrast-enhanced CT images, and the patient-level classification performance of the ViT architecture was evaluated in particular. The results revealed that the ViT model achieved higher Accuracy (89.19%), F1-score (86.67%), and area under the curve (AUC) with 0.946 than other CNN and transformer-based models. These results demonstrate that transformer-based architectures have a stronger representational capacity for distinguishing pathological changes in pancreatic CT images.

In this study, a Wilcoxon signed-rank test was applied to evaluate the statistical significance of the performance difference between the proposed ViT model for classification of AP and normal pancreas from CT scans and other state-of-the-art models. The test was performed using the classification accuracy results for the test set (37 patients) (correct = 1, incorrect = 0). The significance level was set at *p* = 0.05. As shown in Table 6, the Wilcoxon signed-rank test results revealed that the ViT model performed statistically significantly better than the EfficientNet and Swin Transformer models (*p* = 0.031). However, the difference in performance between the ViT model and the ConvNeXtV2 (*p* = 0.109) and ResNet50 (*p* = 0.068) models was not statistically significant. These results demonstrate that the ViT architecture provides better, reliable performance in classifying pancreatic CT images due to its ability to model global contextual features.

In this study, 95% confidence intervals (CI) for the classification accuracy of deep learning models were calculated using Bootstrap analysis as shown in Table 7. The ViT model achieved an accuracy rate of 89.19% (95% CI = 75.3–96.4%), demonstrating higher performance than all other models. Furthermore, odds ratio values between the ViT model and the other models were calculated using an effect size analysis. The results of this analysis show that the ViT model has clinically significant performance in terms of odds ratio values when compared to other methods.

Overall, the enhanced performance of the transformer-based ViT architecture can be explained by its global contextual feature learning capability provided by its self-attention mechanism. Unlike CNN-based architectures, which generally focus on local feature extraction, transformer architectures can model long-range relationships between different regions within an image. This feature enables more accurate learning of complex pathological findings, such as inflammation, edema, and changes in tissue density in pancreatic tissue. ROC analysis results also support this, with the ViT model having the highest AUC value. This indicates that the model performs stronger discriminative performance across different threshold values. These results show that the ViT architecture is a powerful alternative for solving medical image classification problems and can be used as an effective decision support system for the automated diagnosis of critical diseases, such as acute pancreatitis.

**Figure 7:**
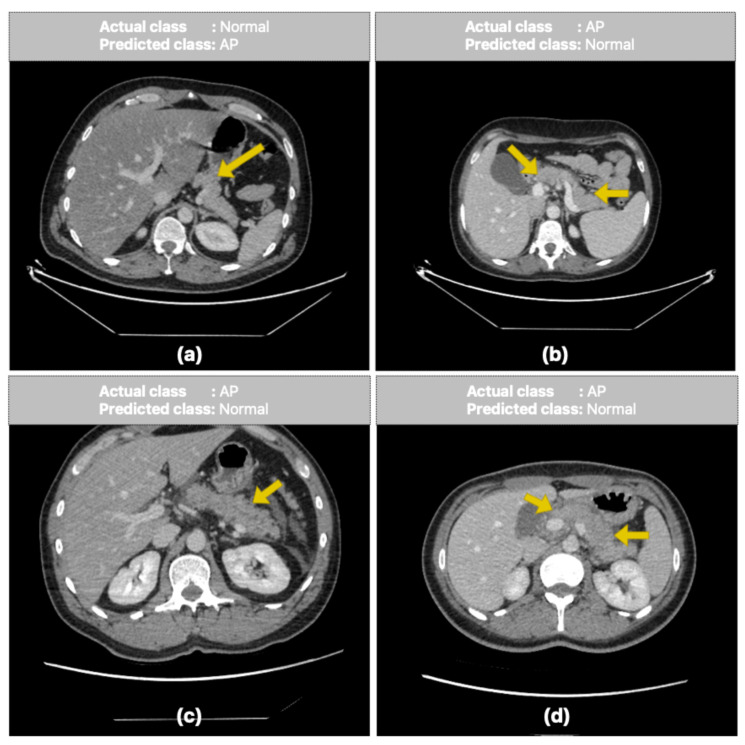
Representative misclassified CT slices in patient-level classification using the ViT, illustrating challenging cases of AP and normal pancreas differentiation. (**a**) false positive case (normal pancreas classified as AP); (**b**–**d**) false negative cases (AP classified as normal pancreas). Yellow arrows indicate regions of parenchymal heterogeneity and wall irregularities.

Figure 7 shows sample CT slices of patients that were misclassified by the ViT-based, patient-level classification approach. In Figure 7a, a patient with normal pancreatic tissue being classified as having AP by the proposed model. This may be due to the model confusing parenchymal density heterogeneity, which is particularly prevalent in the corpus and tail regions of the pancreas, with inflammatory changes. By contrast, in Figure 7b–d, patients diagnosed with AP were classified as normal. In these false-negative cases, the expected heterogeneity in the pancreatic parenchyma was not sufficiently prominent due to the images being acquired in the venous phase. In addition, the presence of free air around the liver and gallbladder alongside pancreatitis findings in some sections is noteworthy and is considered potentially related to perforation of the hollow viscus. Such complex, concomitant pathologies can make it difficult for the model to distinguish features, leading to misclassifications. Overall, these findings suggest that the model has difficulty with borderline cases, situations involving low contrast differences and cases with concomitant abdominal pathologies. Such cases are also clinically challenging from a diagnostic perspective.

In this study, the classification of normal pancreases and AP was primarily performed at the patient-level by evaluating CT images containing the pancreas. In addition, data split technique was also employed to prevent data leakage between the training and test sets in patient-level classification. Therefore, patient-level classification was obtained by aggregating slice-level predictions, reducing the impact of misclassified individual slices and providing a more stable, clinically meaningful decision. This significantly increases the number of training and test samples, despite the limited number of patients. Although the number of patients with AP may appear limited in patient-level classification, several factors support the reliability of the proposed classification framework. Each patient contributes a large number of CT slices, enabling the model to recognize a variety of anatomical and pathological patterns. Furthermore, the dataset includes patients with varying degrees of AP and different clinical presentations, enhancing the robustness of the model by providing heterogeneity. To mitigate potential limitations arising from the size of the dataset, data augmentation techniques were also applied during training to improve generalization and reduce overfitting.

The clinical applicability of AI systems in medical imaging goes beyond model performance and depends on several critical factors, including data quality, generalizability and integration into clinical workflows. While deep learning models, particularly transformer-based architectures, have demonstrated encouraging outcomes in medical image analysis, their implementation in the real world remains challenging. One important aspect is data quality and representativeness. In this study, the dataset was collected from a single center and reflects real-world clinical conditions, including variations in imaging protocols and patient characteristics. Furthermore, translating AI models into clinical practice requires robust validation across multicenter datasets, standardized imaging protocols and prospective evaluation. The variability encountered in routine clinical settings may not be fully captured by models trained on retrospective datasets. Another key consideration is interpretability and clinical acceptability. While the proposed model shows promise, its adoption in clinical workflows would require explainability mechanisms and integration with radiological decision-making processes.

## 5. Conclusions

This study comparatively evaluates CNN and Transformer-based deep learning architectures for the automatic classification of AP and normal pancreas from contrast-enhanced CT images. Within the proposed system, ResNet50, EfficientNet, ConvNeXtV2, Swin, and ViT architectures were analyzed both on a slice-by-slice and patient-by-patient basis. One of the most significant contributions of the study is its approach to the AP classification problem using a patient-level assessment method, which is more compatible with clinical decision-making processes. Furthermore, dividing the dataset into patient-level training and test sets prevented data leakage, allowing for a more reliable evaluation of model performance.

The experimental results revealed that transformer-based architectures performed better than CNN-based models, particularly in the patient-by-patient classification task. The ViT model achieved the highest overall performance with an 89.19% patient-by-patient Accuracy rate and a 0.946 AUC value. These results demonstrate that the ViT architecture can more effectively model pathological changes in pancreatic tissue thanks to its self-attention mechanism. Although the Swin model achieved the highest accuracy rate in slice-level analysis, the ViT model was observed to exhibit more balanced and robust classification performance in patient-level evaluation. This finding suggests that transformer-based architectures can provide more reliable results, particularly at the clinical decision-making level.

Another important contribution of this study is its patient-level approach to the AP classification problem, which is different from existing studies in the literature. Most existing studies focus on slice-level classification, which may not provide directly applicable results in clinical practice. The patient-level evaluation approach used in this study increases the usability of the model as a decision support system in clinical settings. However, the study has some limitations. Firstly, the single-center nature of the dataset and the relatively small test dataset may affect the model’s generalizability. Secondly, the study used 2D slices and using 3D CT volumes could improve model performance further. Thirdly, model performance could be evaluated using datasets obtained from different imaging protocols and devices. Future studies could evaluate the generalizability of transformer-based architectures using larger, multicenter datasets. In addition, using 3D deep learning architectures and integrating time series analyses could improve model performance further. Moreover, integrating explainable AI methods could make model decisions more clinically interpretable.

## Figures and Tables

**Table 1:** Demographic distribution of the study population including AP and normal groups.

		Acute Pancreatitis (AP)		Normal		Total	
Variable		*n*	%	*n*	%	*n*	%
Gender	Female	45	56.25	35	34.0	80	43.7
	Male	35	43.75	68	66.0	103	56.3
		Mean	SD	Mean	SD	Mean	SD
Age	Female	59.8	17.0	44.4	12.1	53.1	16.8
	Male	56.0	13.5	39.9	13.8	45.4	15.7

SD: Standard deviation, *n*: Number of patients.

**Table 2:** Biochemical and hematological characteristics of patients in the acute pancreatitis group.

		Female (*n*: 45)			Male (*n*: 35)		
Parameter	Unit	Mean	Median	SD	Mean	Median	SD
**Amylase**	U/L	1578.05	1355.35	958.65	1338.56	1152.50	1182.88
**Lipase**	U/L	3650.51	2837.50	2523.62	2871.63	1834.90	2829.78
**GGT**	U/L	280.43	125.00	414.39	258.21	200.00	220.12
**Urea**	mg/dL	32.09	30.00	10.37	30.74	31.00	10.19
**Creatinine**	mg/dL	0.77	0.77	0.18	0.96	0.98	0.16
**WBC**	×10^3^/µL	12.43	11.33	4.85	12.91	12.86	4.80
**Neu#**	×10^3^/µL	10.26	8.61	4.91	10.35	10.45	4.77
**Platelet Count**	×10^3^/µL	277.95	263.00	68.98	245.62	249.00	75.43
**Hemoglobin**	g/dL	13.00	13.05	1.89	14.43	14.85	2.04
**Hematocrit**	%	38.70	38.70	5.01	42.32	42.75	5.18
**CRP**	mg/L	9.25	4.55	12.88	32.51	11.25	50.79

GGT: Gamma-Glutamyl Transferase, WBC: White Blood Cell Count, Neu#: Neutrophil Count, CRP: C-Reactive Protein.

**Table 3:** Comparative performance analysis of deep learning models for patient-level classification.

Model	Precision (%)	Recall (%)	F1 (%)	Accuracy (%)
ResNet50	83.33	62.50	71.43	78.38
EfficientNet	73.33	68.75	70.97	75.68
ConvNeXtV2	80.00	75.00	77.42	81.08
Swin	83.33	62.50	71.43	78.38
ViT	92.86	81.25	86.67	89.19

**Table 4:** Patient-level classification performance of deep learning models with data augmentation.

Model	Precision (%)	Recall (%)	F1-Score (%)	Accuracy (%)
ResNet50	58.33	82.35	68.29	76.58
EfficientNet	61.46	89.40	72.84	80.18
ConvNeXtV2	67.71	76.47	71.82	77.03
Swin	79.17	85.40	82.16	85.14
ViT	72.92	90.90	80.92	85.14

**Table 5:** Slice-level classification performance comparison of deep learning models.

Model	Precision (%)	Recall (%)	F1-Score (%)	Accuracy (%)
ResNet50	86.78	69.75	77.34	80.07
EfficientNet	82.20	71.53	76.50	78.57
ConvNeXtV2	85.23	71.13	77.54	79.91
Swin	93.78	72.10	81.52	84.06
ViT	85.13	80.78	82.90	83.75

**Table 6:** Wilcoxon signed-rank test results comparing ViT with other models.

Model Comparison	ViT (Correct)	Model (Correct)	Number of Effective Pairs	*p*-Value
ViT vs. EfficientNet	33	28	7	0.031
ViT vs. ConvNeXtV2	33	30	3	0.109
ViT vs. Swin	33	29	6	0.031
ViT vs. ResNet50	33	29	4	0.068

**Table 7:** Classification accuracy, 95% CI, and odds ratio effect size for model comparison.

Model	Accuracy (%)	95% CI	Odds Ratio vs. ViT
ViT	89.19	[75.3–96.4]	-
ConvNeXtV2	81.08	[65.0–91.2]	1.52
ResNet50	78.38	[62.8–89.2]	2.28
Swin	78.38	[62.8–89.2]	2.28
EfficientNet	75.68	[59.7–87.6]	2.79

## Data Availability

The data presented in this study are available on request from the corresponding author due to ethical restrictions.
